# Realtime Gas Emission Monitoring at Hazardous Sites Using a Distributed Point-Source Sensing Infrastructure

**DOI:** 10.3390/s16010121

**Published:** 2016-01-20

**Authors:** Gianfranco Manes, Giovanni Collodi, Leonardo Gelpi, Rosanna Fusco, Giuseppe Ricci, Antonio Manes, Marco Passafiume

**Affiliations:** 1 Department of Information Engineering (DINFO), Università di Firenze, via Santa Marta 3 50139 Firenze, Italy; giovanni.collodi@unifi.it (G.C.); marco.passafiume@unifi.it (M.P.); 2 Eni Spa; Piazzale Enrico Mattei, 1 00144 Roma, Italy; leonardo.gelpi@eni.com (L.G.); rosanna.fusco@eni.com (R.F.); giuseppe.ricci@eni.com (G.R.); 3 Netsens Srl, via S.Pertini 93 50019 Sesto Fiorentino (Fi), Italy; antonio.manes@netsens.it

**Keywords:** VOC monitoring, wireless sensor networks, photoionization detectors

## Abstract

This paper describes a distributed point-source monitoring platform for gas level and leakage detection in hazardous environments. The platform, based on a wireless sensor network (WSN) architecture, is organised into sub-networks to be positioned in the plant’s critical areas; each sub-net includes a gateway unit wirelessly connected to the WSN nodes, hence providing an easily deployable, stand-alone infrastructure featuring a high degree of scalability and reconfigurability. Furthermore, the system provides automated calibration routines which can be accomplished by non-specialized maintenance operators without system reliability reduction issues. Internet connectivity is provided via TCP/IP over GPRS (Internet standard protocols over mobile networks) gateways at a one-minute sampling rate. Environmental and process data are forwarded to a remote server and made available to authenticated users through a user interface that provides data rendering in various formats and multi-sensor data fusion. The platform is able to provide real-time plant management with an effective; accurate tool for immediate warning in case of critical events.

## 1. Introduction

The application of wireless sensor network (WSNs), complemented by low-cost, low-power consumption gas sensors, has received considerable attention in the last several years [1,2,3]. Volatile Organic Compounds (VOCs) are widely used in industry as solvents or chemical intermediates. Unfortunately, they include components that, if present in the atmosphere, may present a risk to human health. VOCs also are found as contaminants or by-products in many processes, such as in combustion gas stacks and groundwater clean-up systems. Therefore, detection of VOCs at sub-parts per million (ppm) levels is of paramount importance for human safety, and, consequently, critical for industrial hygiene in hazardous environments [4,5]. The most commonly used portable field instruments for VOC detection are hand-held Photoionization Detectors (PIDs), which may be fitted with pre-filter tubes for detection of specific gases. Recently, wireless hand-held PIDs have become available on the market, thus providing ubiquitous operation, but they have a limited battery life and are relatively costly. 

Oil and gas activities also can be associated with releases of pollutants that, although at low concentrations are not considered toxic to human health, have a very low odor threshold and can have a significant effect, causing complaints from neighboring populations. Most of these pollutants have an odor threshold below analytical detectability and cannot be monitored directly at these levels by portable detectors; however, others, like hydrogen sulphide (H_2_S), can be detected with detectors similar to those used for VOCs. Together with VOC detection, H_2_S monitoring can provide real-time information about the potential odorous effects of a plant’s activities, helping operators to manage critical situations quickly. 

This paper describes the implementation and on-field results of an end-to-end, distributed monitoring system using VOC and/or H_2_S detectors and capable of performing real-time detection of gas emissions at potentially hazardous sites at a per-minute data rate [1,3,6].

A heavy problem for this class of distributed systems is the need for periodic calibration or substitution of PID sensors distributed over large observation areas: in this paper an innovative concept of calibration management is presented, by which also non-specialized maintenance operators can perform calibration routines for every PID only acting on the single sensor, without the need of any kind of intervention on the core of the network thus leading to a better overall self-maintained reliability. By this, the paper discusses and demonstrates an original physical model of the PID. The model allows a circumvention of a major limitation in calibration at low parts per billion (ppb), at which the PID characteristic is non-linear. The model predicts the PID characteristic on the basis of the physical parameters of the PID family, allowing the characteristic to be linearized. On that basis, PID calibration can be performed using the standard zero/span procedure over the entire ppb-ppm range. 

The system consists of a WSN infrastructure with nodes equipped with weather-climatic sensors and VOC detectors and fitted with TCP/IP over GPRS (TCP/IP over General Packet Radio Services, Internet Protocols over mobile networks) gateways to forward the sensor data via the internet to a remote server. The continuous monitoring of benzene emissions from a benzene storage tank is demonstrated, using a unique wired/wireless configuration installed in ATEX Zone 0 (or rather in an hostile/hazardous environment, following definition given in the European 94/9/EC directive: Appareils destinés à être utilisés en ATmosphères EXplosibles [6]). 

As previously described, the first system was installed in a petrochemical plant in Mantova, Italy, where it has been in continuous, unattended operation since April 2011. A second, wider system including H2S detectors in addition to VOC detectors was installed in October 2014 at Gela Refinery (RAGE) and will be described in detail.

## 2. RAGE Installation Overview

**Figure 1:**
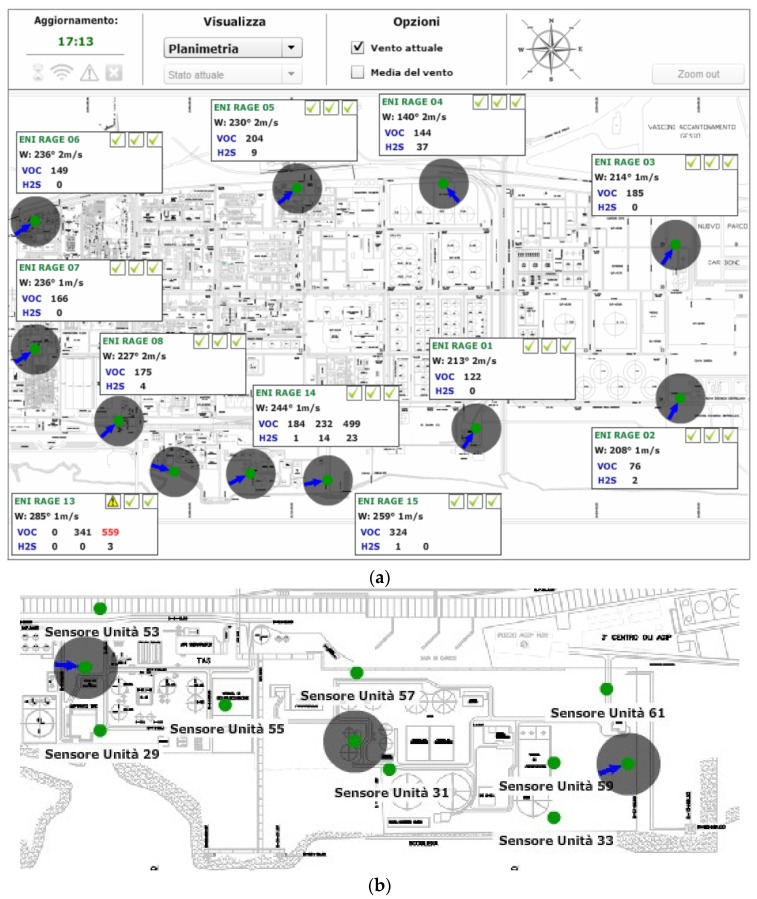
(**a**) layout of the RAGE installation; (**b**) close-up of SNU and end node units (ENU) deployment around the wastewater treatment plant.

Figure 1a represents the RAGE distributed point-source infrastructure, which consists of 11 main Sink Node Units (SNUs) equipped with air temperature/humidity sensors and an anemometer connected to a central server via TCP/IP over GPRS. 

Representative locations were identified along the perimeter of the industrial area, along with several specific internal sites where odours and hazardous emissions might occur (as shown in Figure 1a,b). Each SNU is connected to one or more wireless End Node Units (ENUs) equipped with a VOC and an H_2_S detector, appropriately distributed across the plant area. This modular approach allows the system to be expanded and/or reconfigured according to specific monitoring requirements, while providing redundancy in case of failure of one or more SNUs.

Owing to the extent and complexity of the RAGE plant, which covers some 300 acres and features complex metal infrastructures, it was suitable to subdivide the area involved into various sub-areas. Each sub-area is covered by a sub-network consisting of a SNU equipped with weather-climatic sensors, such as wind speed/direction (WSD) and air relative humidity/temperature (RHT) (ENI 1‒ENI 7 in Figure 1), and ENUs equipped with VOC and H_2_S detectors. In addition, the RAGE 2 unit is equipped with a rain gauge and solar radiation sensor. 

Furthermore, when all weather-climatic measurements are collected, they provide a map of the relative RHT and WSD in the area, which are crucial to providing accurate VOC-sensor readout compensation [7]. The need for so many wind stations across the plant property is justified by the area’s turbulent wind distribution, which can be observed from the various orientations of the blue arrows representing wind direction in Figure 1.

Figure 1b represents the layout of the sub-network deployed around the wastewater treatment plant. The sub-network consists of three SNUs equipped with weather sensors (air/wind), each connected to two or three ENUs spaced at tens of meters apart. Sampling the VOC/H_2_S concentration at intervals of tens of meters allows evaluation of VOC emissions dispersion; in addition, information about WSD allows accurate estimation of the emission’s source.

VOC/H_2_S concentration and weather-climatic data are updated every minute; however, data are collected every 10 s and averaged prior to transmission to the SNU. This intensive sampling interval allows accurate assessment of the evolution of gas concentrations. In fact, owing to the rapid variability of wind direction/intensity determined by local turbulences, the value of the gas concentration varies very rapidly. 

Furthermore, an intensive sampling interval helps the operator to be aware in real time about any critical situation occurring in the plant. With this aim, the system provides the opportunity to fix threshold concentration values (customized for each node of the grid) and to set an automatic e-mail alert when those threshold values are exceeded.

### 2.1. The Communication Platform

For achieving improved flexibility and scalability, wireless communications between the ENUs and their owner SNUs (performed in the unlicensed ISM UHF band @868 MHz) rely on a standard TCP/IP protocol on which was added a further MQTT-like routing communication layer [8]. Inside every communication packet, each ENU sends an unique ID code configurable with selectors on ENU hardware board. When the SNU receives sensor data, the data is contextualized to the position of the specific ID-th ENU: its related information is stored in specific routing tables shared between Internet connected servers and the network of SNUs which relate the sensor ID with its position and sensing capabilities. 

The implementation of a simplified routing layer over the standard TCP/IP stack allows system administrators or automatic DHCP (Dynamic Host Configuration Protocol) services to dynamically change the IP for each ENU, if it is needed for any reason, without any kind of data connection loss.

A further heavy problem for bigger VOC sensor networks management is the taking into account of calibration issues for each sensor in the network. VOC sensor technology is known to have time-limited sensing life (for example, due to progressive degradation of implemented UV lamp), so an automatic routine of progressive calibration correction as far as the simplification of the degraded sensor components replacement procedure are of extremely usefulness: for this, in a proposed proprietary protocol single ENU–SNU data packets also embed the ID-th ENU calibration values, like sensitivity parameters or offset correction values. Thus, ENU can perform different kinds of automatic recalibration routines without the need of a direct manual reconfiguration. In addition, when sensor component replacement occurs, the non-specialized maintenance operators will only have to set new calibration parameters for the specific ENU without having to intervene in other settings.

Communication between SNUs and Internet connected servers rely on a GSM mobile-network featuring a TCP/IP protocol with DHCP, providing Internet connectivity. To avoid policy management of mobile network service provider on IP assignments, and to give the maximal network expandability, a VPN (Virtual Private Network, using specific GSM SIM cards) will be implemented in the future: for now, the application of the MQTT-like routing scheme [8] over the TCP/IP stack easily resolve any IP management issue.

#### 2.1.1. The SN and WI Units

The SNU consists of a GPRS antenna, a GPRS/EDGE quadriband modem, a sensor board, an Input/Output (I/O) interface unit and an ARM-9 micro-controller operating at 96 MHz. The system is based on an embedded architecture with a high degree of integration among the sub-systems.

The unit is equipped with various interfaces, including LAN/Ethernet (IEEE 802.1) with TCP/IP protocols, USB ports and RS485/RS422 standard interfaces. The sensor board is equipped with eight analogue inputs and two digital inputs. The SNU also is equipped with a wireless interface (WI), which provides wireless connectivity to the ENUs. 

#### 2.1.2. The ENU

The ENU consists of a VOC sensor board and VOC/H_2_S detectors. The acquisition/communication sub-system of the ENU is based on an ARM Cortex-M3 32-bit micro-controller, operating at 72 MHz, which provides the necessary computational capability on the limited power budget available.

To reduce the power requirement of the overall ENU sub-system, two different power supplies have been implemented, one for the micro-controller and one for the peripheral units.

The microcontroller is able to connect/disconnect the peripheral units, thus conserving local energy resources. The VOC detector sub-system is powered by a dedicated switching voltage regulator; this provides a very stable and spike-free energy source, as required to properly operate the VOC detector.

Communication between the ENU and the VOC detector board is based on an RS485 serial interface, providing high-level immunity to interference and bidirectional communication capability, which is needed for remote configuration/reconfiguration of the unit.

#### 2.1.3. Network Structure and Routing Schemes

From among the alternatives, a hierarchical-based routing scheme was selected based on the particular nature of the installation: the extended area of the plant, the few critical source-areas of potential emissions requiring a dense deployment, and the highly uneven distribution of nodes over the area. As stated previously, the installation was partitioned into sub-networks to be deployed around the critical sites, with one SNU for each individual sub-network. Wireless connectivity between the SNUs could have been implemented, using one specific SNU as a gateway to the Internet. 

However, this option conflicted with at least two of the major requirements. The first is the need for redundancy in case of failure of the gateway unit; in fact, in this scenario, Internet connectivity would be lost, with consequent loss of real-time updating capability, which is considered a mandatory requirement of the system. The second need which would not have been met is that of providing full connectivity among individual SNUs under conditions in which line-of-sight propagation was not guaranteed, due to the presence of such temporary obstacles as trucks or maintenance infrastructures. 

A multiple GPRS gateway overcomes those limitations; even in the case of failure of one or more gateway units, Internet connectivity would be provided by those still in operation, while the issue of the obstacles is circumvented. As for wireless connectivity, a star configuration was preferred to a mesh configuration, given the limited number of nodes and the need to minimize latency.

#### 2.1.4. Protocols and WSN Services

Two levels of communication protocols were implemented, in a mesh network topology. The upper level handles communications between the SNUs and the server using a custom binary protocol on top of a TCP layer. This level was designed and calibrated for real-time, bidirectional data exchange, in which periodic signaling messages are sent by both sides [9]. Since the sensor network necessitates a stable link, quick reconnection procedures for broken links were important. To ensure minimal data loss, the SNUs have non-volatile data storage, as well as automatic data packet retransmission (with timestamps) after temporary downlink events. Furthermore, this design is well suited to low-power embedded platforms like ours, with limited memory and power resources. In fact, our protocol stack currently requires about 24 KB of flash memory (firmware) and 8 KB of RAM.

In contrast to the upper level, the lower one concerns local data exchange between network nodes. Here, a cluster-tree topology was employed; each node, which both transmits and receives data packets, is able to forward packets from the surrounding nodes as needed. 

### 2.2. Energy Budget Issues

Energy budget plays a key role in maintainability of the WSN [4,7,9]. In our case, this is made even more critical by the necessity to provide standalone operation with periodic maintenance intervals exceeding four months.

Since electrical energy from the plant could not be used, secondary sources had to be available locally; photovoltaic panels (PVP) fit the bill. All SNUs are equipped with PVPs, as they must support a number of functions, including connectivity and data collection from sensors. The ENUs, when equipped with low-energy demanding sensors, have three to five years of battery life using primary sources.

However, in this installation, the ENUs must support the power-hungry VOC sensors. For this reason, the ENUs also are equipped with PVPs.

To rely on autonomous energy resources while providing continuous operation, a secondary energy source was integrated into the ENU to supply the 360 mW + 720 mW average power required. A 5-W PVP can fulfil the task only under ideal sunlight conditions, such as in summer, but hardly at all in winter. The PVP unit includes a charge regulator specifically designed to provide maximum energy-transfer efficiency from the panel to the battery under any operating condition. Great attention was paid to the design of the voltage regulator, as the secondary energy source plays a key role in ensuring standalone, unattended operation of the communication platform. 

The overall system has been deployed fully since October 2014; owing to favorable climatic conditions, the energy balance is positive, and operation has continued uninterrupted, even without replacing the batteries periodically, as required in the Mantova plant [1,7].

The VOC sensor energy budget is greater than that needed by the H_2_S detector, which mainly is capacitive, or by the computational/communication unit. This is a critical issue for the ENUs, as the PIDs used for reading the VOC concentration must be powered-on continuously to operate efficiently. The actual current drawn by the PIDs resulted in some 30 mA, corresponding to 720 mAh a day, almost twice the amount required by the communication/computational units, ranging up to 360 mW a day. The ENU’s primary source capacity is 60 Ah, which provides more than 2 full months of continuous operation. 

The H_2_S detector energy budget is very limited, however, as the electrochemical devices are capacitive and absorb a very low standby current, below 1 mA. 

### 2.3. VOC and H2S Detectors

The VOC detector is a key element of the monitoring system’s functionality. For this application, two criteria were considered mandatory. The first is that the VOC detector should be operated in diffusion mode, thereby avoiding pumps or microfluidic devices that would increase energy requirements and make maintainability issues more critical. The second criterion was that the system should be able to operate in the very low-ppb range, with a Minimum Detectable Level (MDL) of some 2.5 ppb with a ±5% accuracy in the 2.5–1000 ppb range, which represents the range of expected VOC concentration. The PID AH provided by Alphasense Ltd. (Nottingham, UK) fulfills most of the requirements [10]. 

However, two major issues that could affect the efficient use of the PID in our system were identified. The first was that in the low-ppb range, the calibration curve of the PID shows a marked non-linearity; this would require an individualized, meticulous, multipoint calibration involving high cost and complexity. The second issue was that when operated in diffusion mode at low ppb and after a certain time in power-off, the detector requires a stabilisation time of several minutes; hence it would not be able to operate at the required one-minute intervals.

Since both of the above-mentioned limitations are intrinsically related to the PID’s physical behaviour, this was investigated carefully, and a behavioral model of the PID was developed to explain these phenomena.

Section 3 introduces an original physical model of the PID, capable of compensating for the non-linearity of the characteristic, thus allowing PID calibration in the overall range, including the low ppbs, using the standard zero/span procedure.

The second issue was circumvented by using the PID always powered-on; this also is consistent with the requirement of sampling gas concentrations at high data rates, as discussed previously.

Combined data analysis of VOC and H_2_S concentrations can help assess odors around the plant and, potentially, in neighboring areas.

## 3. A Physical Model of PIDs

**Figure 2:**
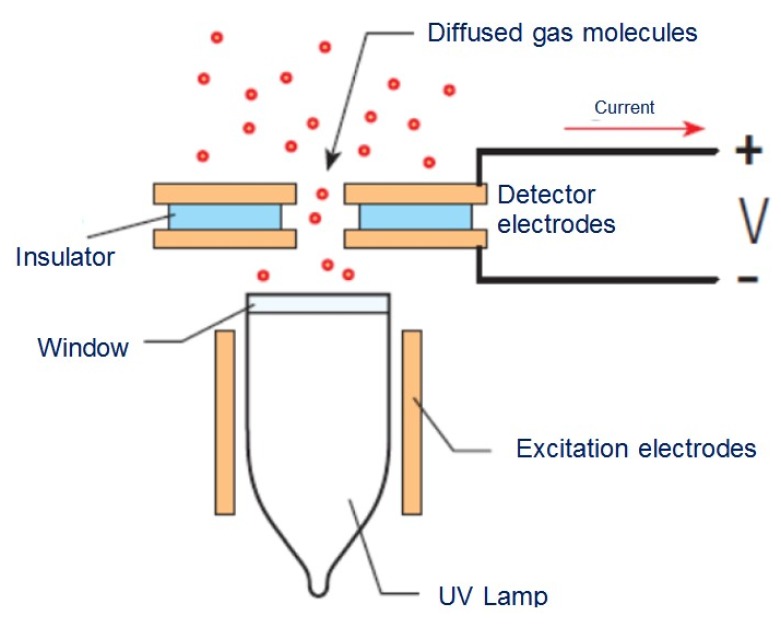
PID schematic.

PIDs are well-known devices that measure VOCs and other toxic gases in low concentrations from ppb up to 10,000 ppm [11,12]. Gas molecule ionization occurring in the PID cell yields a current determined by opposite-polarity ions collected by a pair of electrodes. Figure 2 represents basic PID operation.

**Figure 3:**
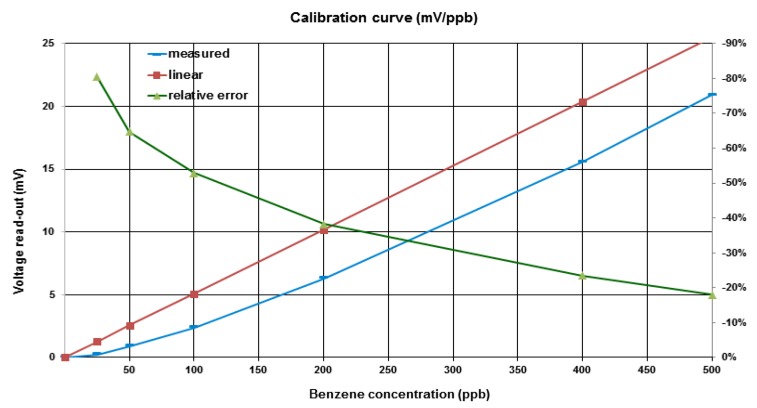
Measured *vs.* linear PID calibration curve and relative error.

Readout current is proportional to gas molecule concentration. In the ppm range, the calibration curve is fairly linear. However, for concentrations in the low-ppb range, the curve is nonlinear, as illustrated in Figure 3, which compares measured readout voltage, the blue line, with the linear curve calculated by PID sensitivity measured at ppm concentration, the red line; the relative error is also represented, by the green line.

The expected concentration level under standard operating conditions measured in the refinery plant is about 100–300 ppb, resulting in an unacceptable error in measuring the concentration. To perform an accurate calibration curve would require time-consuming, costly multipoint calibration for each PID. Considering that the projected PID lifetime under continuous operation is six months, such calculations would result in an unacceptable service cost. Therefore, the PID’s physical behavior was investigated to derive a closed-form equation of the calibration curve, with the aim of performing the calibration by standard zero/span gas measurements [13]. 

### 3.1. PID Behavioral Model in Steady-State Conditions

As is well known, the photoionization effect implies the absorption of a photon, with consequent ionization of the molecule according to the relationship:

(1) $$RH+\hbar \nu \to RK^{+}+e^{-}$$

where RH is the molecule and *hν* is the photon energy higher than the molecule ionization potential.

**Figure 4:**
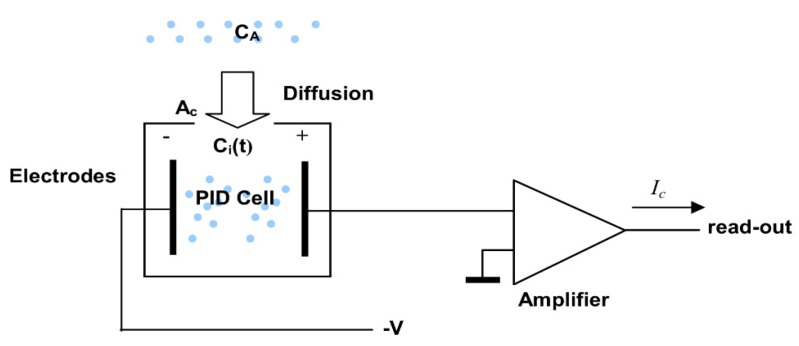
Ionization and diffusion process under steady-state conditions.

Under steady-state operating conditions, a balance is established between the reduced molecule amount due to the ionization process and the flow of molecules from the external environment. The flow is generated by the concentration gradient established both inside and outside the cell, owing to the ionization process, as represented in Figure 4, where *C_A_* and *C_i_* are the external and internal concentrations, respectively.

First, we calculate the molecule flow due to the concentration gradient, that is, the number of molecules flowing in the same time unit through the section *Ac*. Second, we calculate the ionization rate, that is, the number of molecules ionized in the time unit. In the steady-state, the two phenomena should be balanced.

#### 3.1.1. Molecule Flow

The analyte diffusion flow, *J*, is represented by Fick’s first law:

(2) $$J=\frac{1}{A_{C}}\frac{\partial n(t)}{\partial t}=-D\frac{\partial C}{\partial x}$$

where *n(t)* [*mol·m*^−2^*·s*^−1^] is proportional to the molecule number entering the PID cell through section A_c_, C is the gas concentration [*mol·m*^−2^] and *D* is the diffusion coefficient, [*m*^2^*·s*^−1^].

**Figure 5:**
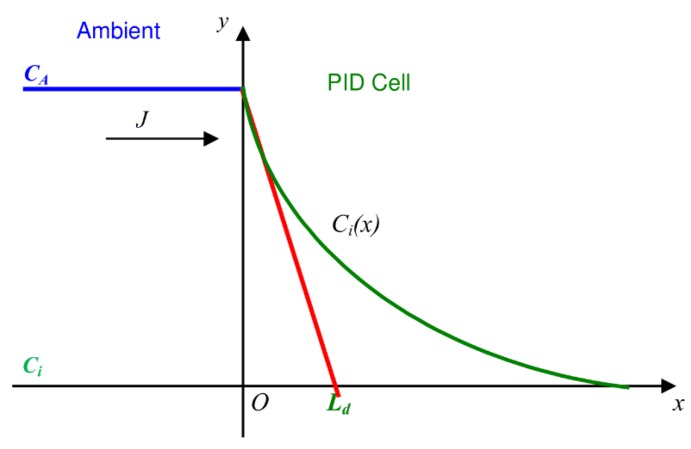
Concentration gradient.

With reference to Figure 5, J can be evaluated at x = 0 as:

(3) $${J(t)|}_{x=0}=\frac{1}{A_{C}}{\frac{\partial n(t)}{\partial t}|}_{x=0}=-D{\frac{\partial C}{\partial x}|}_{x=0}$$

that is, the molecule flow is proportional to the concentration gradient established both outside and inside the cell.

From Fick’s second law, it also can be shown that:

(4) Ci(x)=[CA−Ci(0)]exp(−xLd)

with *L_d_* diffusion length, and

(5) $${\frac{\partial C(x)}{\partial x}|}_{x=0}=-\frac{C_{A}-C_{i}(0)}{L_{d}}$$

Considering: C**_A_** is External concentration, C(x) is Concentration inside the cell at distance x, C_i_ is Steady-state concentration inside the cell, D is Gas diffusion co-efficient, J is Analyte flow, L_d_ is Analyte diffusion length, V_c_ is Cell volume.

Replacing Equation (5) in Equation (3) and letting C_i_(0) = C_io_ yields:

(6) $$J_{D}=\frac{1}{A_{C}}{\frac{\partial n}{\partial t}|}_{x=0}=D\frac{\left(C_{A}-C_{io}\right)}{L_{d}}$$

Stated *V_c_*, the cell volume, the Equation (6) can be rewritten as:

(7) $$\begin{array}{c} {\frac{\partial C_{i}}{\partial t}|}_{x=0}=\frac{A_{C}D\left(C_{A}-C_{io}\right)}{V{\text{​}}_{C}L_{d}}=\frac{L_{d}A_{c}}{V_{c}}\frac{\left(C_{A}-C_{io}\right)}{\tau_{D}}=\frac{C_{A}-C_{io}}{{\tau_{D}}^{*}}\\ \text{with }L_{d}=\sqrt{D\tau_{D}};\;{\tau_{D}}^{*}=\frac{V_{C}}{L_{d}A_{c}}\tau_{D} \end{array}$$

where Ld is the diffusion length in meters, D is the diffusion coefficient in m²/s and $\tau_{D}$ is the rate in seconds at which the gas molecules flow in the cell, normalized to the cell size.

#### 3.1.2. Ionization Time

Letting *I_o_* (incident photons per second) be the photon flow emitted by the UV lamp, the number of ionizations for a time unit is given by:

(8) $$\frac{\partial n(t)}{\partial t}=K\cdot I_{n}\cdot n_{T}\frac{\sigma }{S}$$

where: I_n_ is number of incident photons per second, n_T_ is molecule number, K is UV lamp efficiency, I_o_*=* K I_n_, σ is molecule cross-section, S is photon beam cross-section, V_c_ is cell volume.

Dividing both members by V_c_, volume of the cell, yields:

(9) $$\frac{\partial C_{i}(t)}{\partial t}=K\;I_{n}\frac{\sigma }{S}C_{i}=K\frac{C_{i}}{\tau_{i}}=\frac{C_{i}}{\tau_{I}}$$

where τI=τiK can be interpreted as the mean time between ionizations.

#### 3.1.3. Equilibrium Equation

The equilibrium equation calls for:

(10) $$\frac{\partial C_{i}}{\partial t}={\left[\frac{\partial C_{i}}{\partial t}\right]}_{D}-{\left[\frac{\partial C_{i}}{\partial t}\right]}_{I}$$

In steady-state conditions, the former becomes:

(11) $$\frac{C_{A}-C_{i}}{{\tau_{D}}^{*}}=\frac{C_{i}}{\tau_{I}}$$

Solving by C_i_ yields:

(12) $$C_{i}=\frac{\tau_{I}}{{\tau_{D}}^{*}+\tau_{I}}C_{A}=\alpha C_{A}$$

(13) $$\alpha \left(C_{A}\right)=\frac{\tau_{I}}{{\tau_{D}}^{*}+\tau_{I}}$$

Equation (12) shows that the ratio between the external and internal concentration depends on the values of the diffusion and ionization times. If the diffusion time $\tau_{D}$ is much lower than ionization time $\tau_{I}$, α tends to 1 and the concentration measured inside the cell equals the ambient concentrations. This is because the ionized molecules are replaced easily by the molecules flowing from outside, keeping pace. Likewise, if the ionized molecules cannot be replaced completely, the concentration inside the cell falls until an equilibrium is reached. The factor α is a function of both C_A_ and *C_i_*:

(14) α(CA,Ci)=CiCA

Assuming the diffusion time to be inversely proportional to the external concentration C_A_, $\tau_{I}$ to be invariant, and letting C_o_ be proportionally constant with the dimension of a concentration, yields:

(15) $$\frac{{\tau_{D}}^{*}}{\tau_{I}}=\frac{C_{o}}{C_{A}}$$

Replacing Equation (15) in Equation (13) yields:

(16) α=11+τD*τI=11+CoCA=CACA+Co

Equation (16) shows that when C_A_ equals C_o_, then α = 0.5, that is, the concentration inside the PID cell equals 50% of the external concentration.

It is convenient to express *α* as a function of C_A_ only. For that purpose, a set of six PID AH supplied by Alphasense Ltd. was measured using facilities available at IMM-CNR in Bologna. Readout measurements were performed at 25, 50, 100, 200, 400, 500, 1000 and 2000 ppbs. The factor α for each individual n-th PID was computed as the ratio between the measured values

(17) αn=(Ci)n=VnSν n=VOUT nSν n⋅G

where *S_vn_* is the n-th PID sensitivity and V_n_ is the n-th PID readout for each measurement.

**Figure 6:**
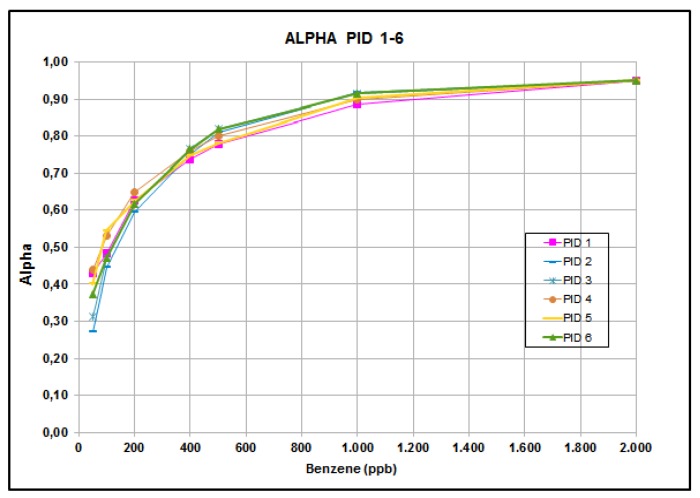
Measured α functions for PID 11-6.

An amplifier is used to achieve current decoupling between the PID itself and the sensing network: by this, V_OUTn_ is the effective voltage readout (next to the amplifier) for the entire sensor and *G* is the known amplifier gain. Figure 6 represents the resulting α functions.

In particular, it can be observed that the concentration value for which C_i_ = 0.5 C_A_ is about 200 ppb and is denoted as C_o_. A PID AH by Alphasense Ltd. [9], fits the requirements of the application described in this paper very well and was used in implementing the monitoring system. Table 1 presents the individual values of C_o_ calculated for each PID; the average value of C_o_ is 118 ppb.

**Table 1:** Calculated *C_o_* for each photoionization detectors (PID).

PID #	1	2	3	4	5	6	Average
S*_v_* (mV/ppm)	153	103.5	51	75	74	51	n.a.
C_o_ (ppb)	118.7	136.6	109.5	116.3	119.5	108.0	

It should be emphasized that C_o_ is independent of the particular value of S*_v_*, which, in turn, is related to *K*, lamp efficiency, and can be regarded as a typical parameter of the specific PID family. Accordingly, the linearization procedure of the calibration curve illustrated previously holds for all devices of the same family. 

The α function fits well to the following expression:

(18) $$\alpha \left(C_{A}\right)=\frac{C_{A}}{C_{A}+C_{o}}$$

**Figure 7:**
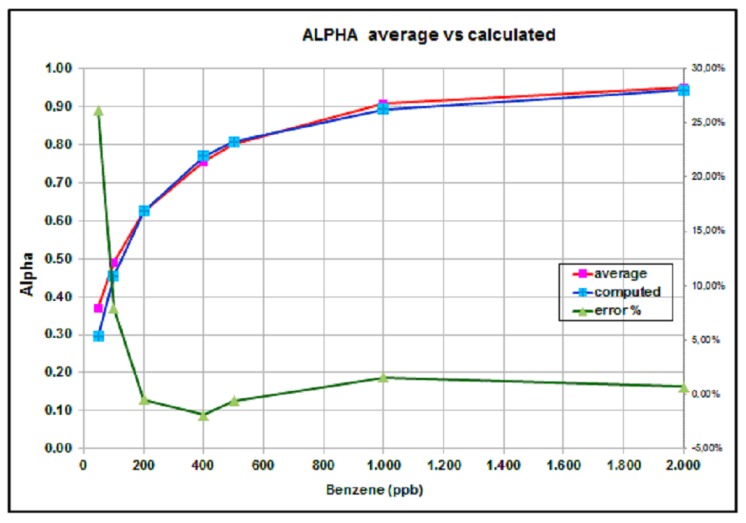
Average, measured α function and relative error percentage.

By averaging the six α functions described previously, one obtains an averaged α function, represented in Figure 7 by the red line.

Comparison between calculated and average α functions in Figure 7 exhibits an excellent match, apart from the 50-ppb value, for which measurement errors probably are relevant.

Taking into account the discussion above, the classical PID zero/span calibration curve should be modified to represent the nonlinear behavior in the low-ppb range, resulting in the following expression:

(19) $$V\left(C_{A}\right)=\alpha {\;S}_{\nu }\;C_{A}=\frac{{C_{A}}^{2}}{C_{A}+C_{o}}S_{\nu }$$

where V(C_A_) is the PID readout in mV and S*_v_* is the PID sensitivity in mV/ppm computed at 5000 ppm isobutylene. Equation (19) represents a generalization of the calibration curve in closed form and allows the calibration curve to be attained with the classical, simple zero/span procedure.

**Figure 8:**
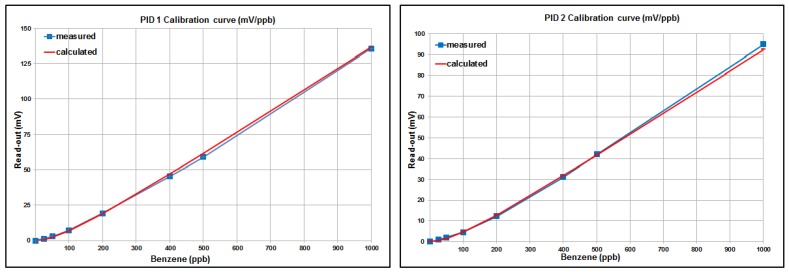

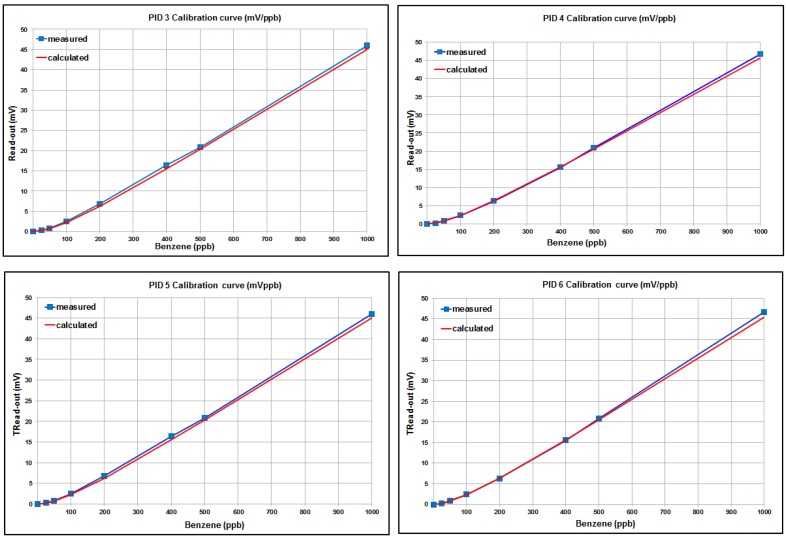
Calibration curves measured and calculated for the set of six PIDs.

To validate the model described previously, in Figure 8, the calibration curves of the set of six PIDs are compared with those calculated on the basis of Equation (19), in the range of 50–1000 ppb, using methane as a reference gas and taking into account the appropriate correction factor.

#### 3.1.4. Linearization Procedure

Rewriting Equation (19) as a function of C_A_ yields*:*

(20) $${C_{A}}^{2}-AC_{A}-AC_{o}=0\text{ with }A=\frac{V\left(C_{A}\right)}{S_{\nu }}$$

Selecting the positive solution of Equation (20) yields:

(21) $$C\left(V_{A}\right)=\frac{A+\sqrt{A^{2}+4AC_{o}}}{2}$$

## 4. Data Rendering and Experimental Results

Data gathered from the field are forwarded to a central database for storage and data rendering. For this purpose, the system has a web-based interface for retrieving and displaying data and for post-processing. The interface features various formats to display the data gathered. It is possible to both access raw data and generate summary reports relating to specific periods and specific network areas. All monitored parameters can be geo-referenced.

Data from individual sensors deployed in the field, either micro-climatic or VOC/H_2_S, can be accessed directly and presented in various formats. 

**Figure 9:**
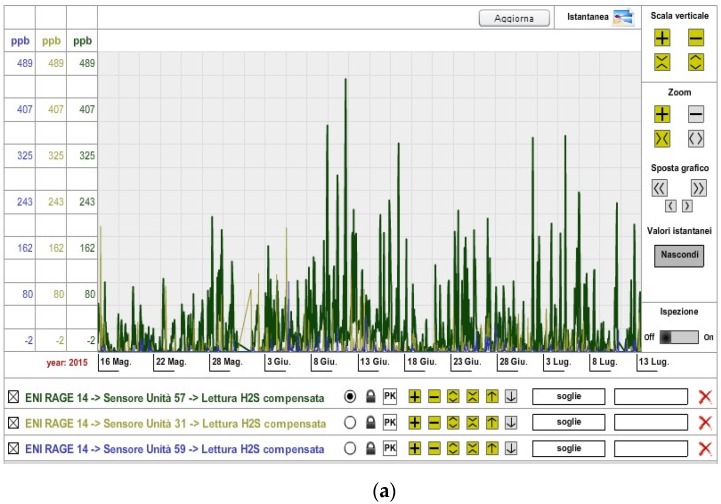

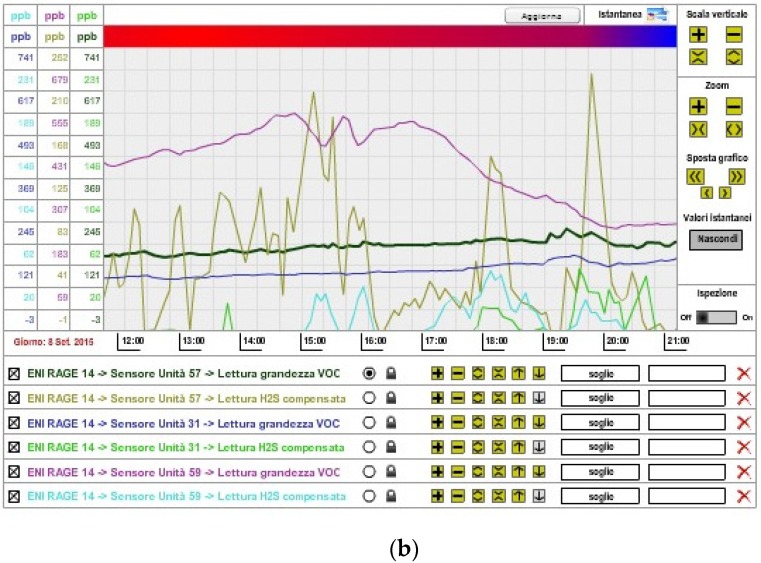
(**a**) VOC and (**b**) H_2_S concentrations in the wastewater treatment plant area.

Figure 9 shows the trend of VOC/H_2_S concentration values detected by the three electrochemical sensors deployed around the wastewater treatment plant over a period of about two months (15 May 2015 to 13 July 2015). Measured values are coherently comparable to each other, demonstrating the effectiveness of the calibration procedure.

### VOC/H2S Concentration and Weather-Climatic Variations

Correlating the microclimatic and wind parameters (air temperature/humidity and WSD) with gas concentrations proved to be very effective for increasing gas readout accuracy and, moreover, for mapping gas concentrations with respect to wind direction, in order to identify possible gas sources.

In fact, when gas sources must be identified, the correlation between WSD and gas concentration is vital. For this reason, a graphic representation that relates these two parameters can be very useful for interpreting results.

**Figure 10:**
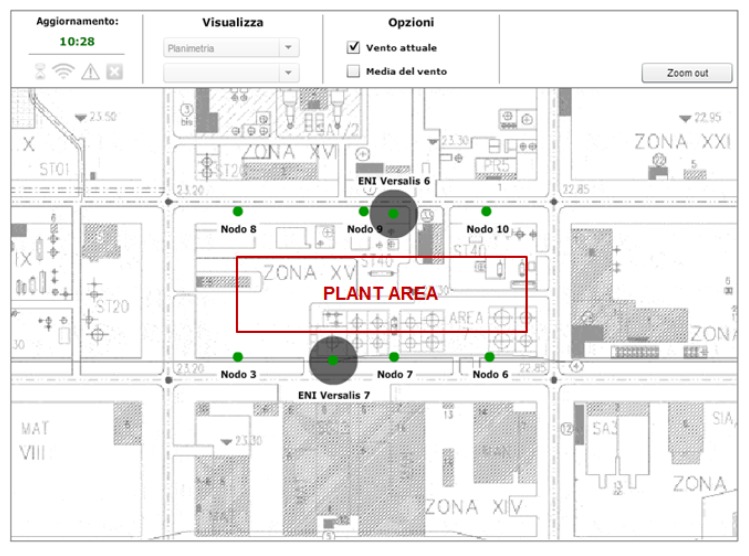
Layout of the six VOC detectors located around a chemical plant in Mantova.

**Figure 11:**
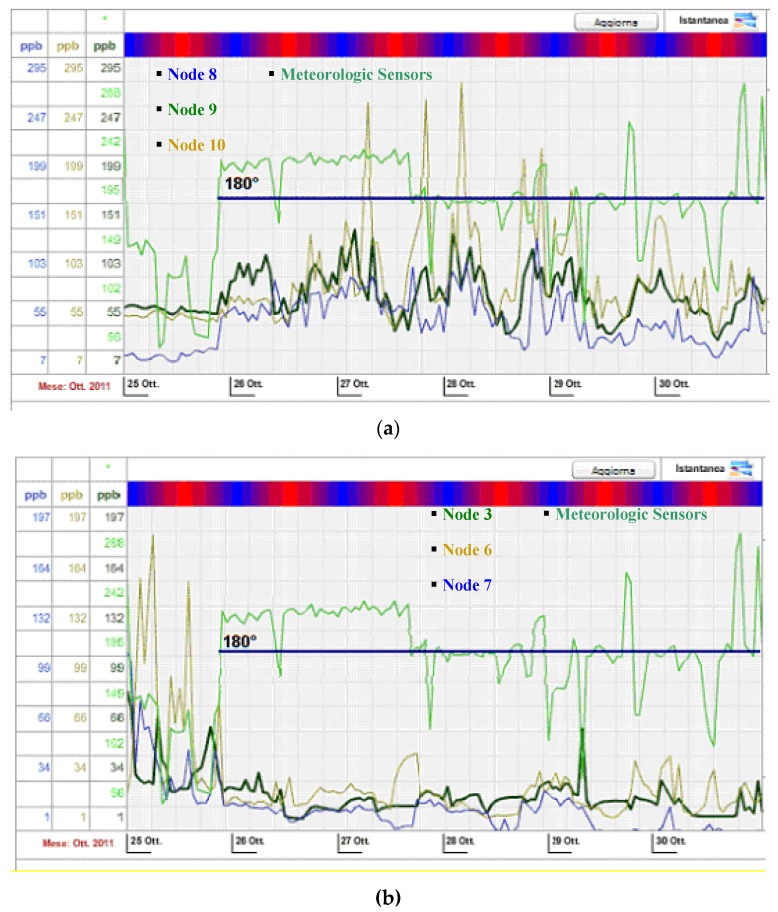
VOC readouts from (**a**) northern side (Sink Node ENI 6) and (**b**) southern side (Sink Node ENI 7) with related wind direction (light green line) on Mantova installation.

The plots in Figure 11, related to the Mantova installation, provide an example of that possibility, in which a suitable VOC detector array is deployed (see Figure 10), with three detectors in a row on both the northern and southern sides of the chemical plant. 

The graph in Figure 11 represents the trend of VOC concentration values (detected by the six PIDs deployed around the chemical plant) over a five-day period.

With reference to the layout of Figure 10, VOC readouts and estimated wind directions from the array on the northern and southern side of the plant are represented in Figure 4: comparing the VOC readouts of the two arrays shows a relationship between traces that is consistent with the south-north direction of the wind and demonstrates the effectiveness of correlating wind direction with speed to identify possible VOC sources. 

Applying these considerations, the system can produce a polar plot of VOC concentration distribution related to wind directions during a given period, providing an overview of the predominant orientation of gas flux during the day.

**Figure 12:**
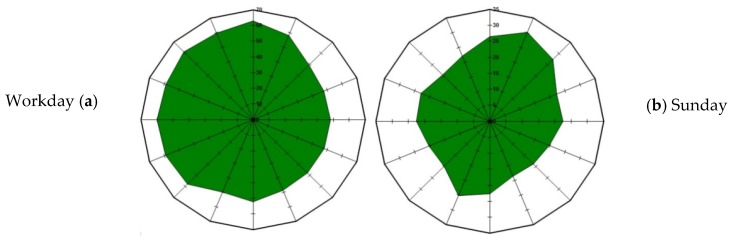
Concentration (radius, ppb) *versus* wind direction (angle) polar plot over 24 h.

To show the polar plot representation usefulness, as an example Figure 12 shows the VOC concentrations in ppb over 24 h during a workday (Figure 12a), and a Sunday (Figure 12b), detected on a point on the western side of the plant.

Due to detector location, concentrations in quadrants I and II of the plot represent the contribution of sources inside the plant while concentrations in quadrants III and IV represent the contribution of VOC sources outside the plant, likely the benzene emissions of vehicles running on the motorway that runs north-south along the western side of plant. When an overall rating of plant VOC emissions is requested, the knowledge of these dynamics results in a far better rating estimation.

**Figure 13:**
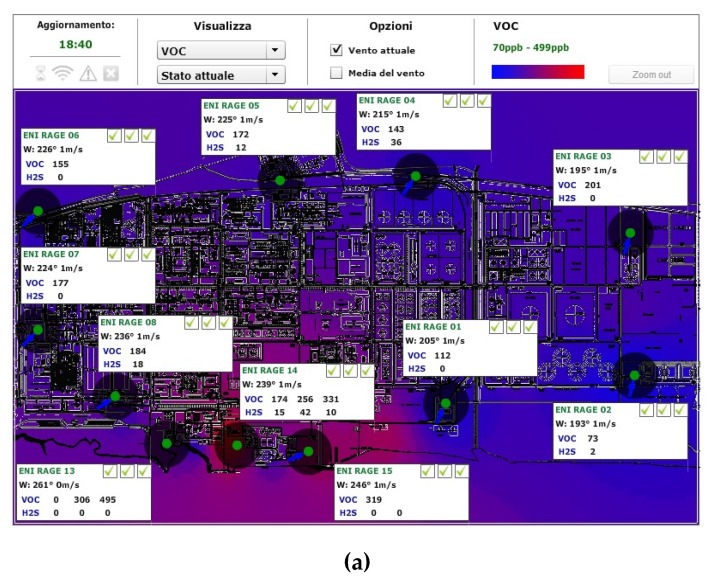

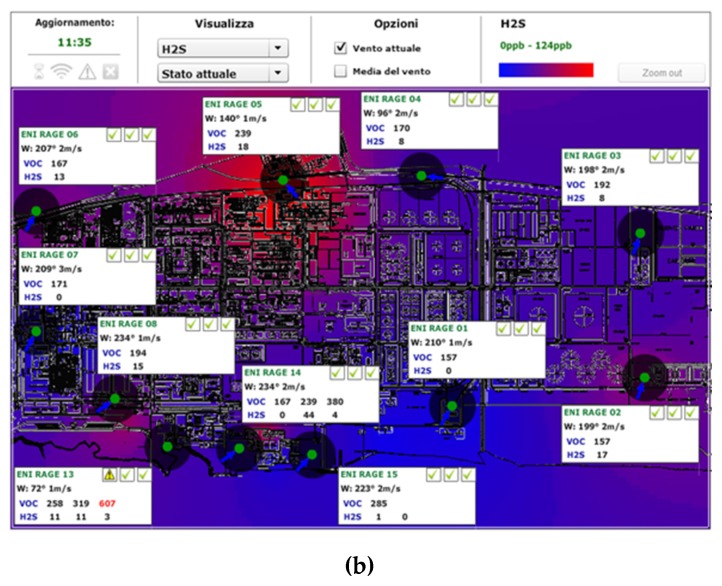
Pseudo-colour map of (**a**) VOC and (**b**) H_2_S concentrations over the RAGE refinery area.

Collected sensor data can also be rearranged to produce pseudo-color maps of VOC/H_2_S concentrations by interpolating readout values detected over the settlement (Figure 13).

## 5. Comparison with State-of-the-Art

This work describes the implementation of a scalable, multi-sensing chemical widespread WSN, initially deployed in the Eni Mantova Chemical Plant and in operation for 6 years; a second application and deployment was done later on in the Gela Eni Refinery since 2014. The aim, in both cases, was to implement a reliable monitoring system capable of supporting the plant security management in early-warning hazardous events, with a minimum of maintenance effort, including ATEX-0 compliant sensors.

Many distributed monitoring application have been described in literature concerning the implementation of WSNs in indoor environments [14,15], also implementing special techniques to reduce sensors power consumption [16], but, in any of them, no stringent requirements have been imposed in terms of reliability, connectivity range and long-term operation in hostile environment. Implementing specific network protocols made by the authors [9,17], all the stringent requirements in terms of reliability, connectivity and maintenance-free life-time (also including the capability of fast-and-simple sensors calibration management) for the imposed scenario are met.

A further key aspect is that the VOC sensors were used in the very low ppb-range, requiring ±5% accuracy. This was achieved by introducing the original PID model, allowing to extend the standard zero-span calibration procedure to the low ppb range, differently from other applications mainly oriented to monitor concentration environments in the ppm range [18,19,20].

VOC sensor application, as far as the implementation of a WSN capable of a reliable early warning and accurate monitoring functionality in the ppb range is not trivial: in [21] a ppb-range, gas real-time monitoring is defined as a “relative challenging task”, and the authors underline the maintenance and calibration complexity and expensiveness for this class of systems. However, some contributions are reported where low ppb range applications are described, using PIDs [22] or CSAs (Colorimetric Sensors Arrays) [23]; the described techniques, indeed, require costly and time-consuming calibration routines for each individual sensor.

In [24], an original approach is described, to achieve a precise gas mixture sensing, based on analysis of the dynamic nonlinear response of a SnO_2_ semiconductor gas sensor; despite the fact that the technique could significantly increase the operational features of gas sensing technology, the required computation complexity could result in corresponding increase of cost-complexity and power consumption of the distributed computational units, thus conflicting with the requirements of a stand-alone system, only relying on autonomous power sources.

This price should be paid when the gas mixture is unknown and the need of resolving the gas composition is mandatory. In our case, however, according to the plant process, VOC and H_2_S are the gas targets needed to define the pollutants concentrations and no gases are present with a significant cross-sensitivity with respect to the target gases. 

For those reasons, no attempt has been made to introduce post-processing techniques to improve sensor sensitivity, while, in other cases, the aforementioned technique could be very useful.

## 6. Conclusions

An end-to-end distributed monitoring system of integrated VOC and H_2_S detectors, capable of performing real-time analysis of gas concentration at hazardous sites on an unprecedented time/space scale, has been implemented and successfully tested at an industrial site. The aim was to provide the industrial site with a flexible, cost-effective monitoring tool to better manage abnormal situations, to identify emission sources in real time, and to collect continuous VOC concentration data using easily re-deployable and rationally distributed monitoring stations.

The piloting of the system allowed us to pinpoint key traits. Collecting data at one-minute intervals meets several needs: identifying short-term, significant events; quantifying the emission effects as a function of weather conditions and of operational process, in addition to identifying potential VOC and H_2_S in the plant area and helping to assess the risk of potential odorous effects. Furthermore, the choice of a WSN communication platform yielded excellent results, above all allowing for redeploying and rescaling the network’s configuration according to specific needs as they arose, while greatly reducing installation costs. Real-time data through a web-based interface allowed both adequate levels of control and quick data interpretation to manage specific situations.

Further work will aim to develop a standard application, allowing deployment of WSN in other plants (such as refineries), and assess potential application of WSN infrastructure monitoring to other environmental indicators.

## References

[1] Manes G., Fusco R., Gelpi L., Manes A., Di Palma D., Collodi G. (2011). Real-Time Monitoring of Volatile Organic Compounds in Hazardous Sites. Environmental Monitoring.

[2] Somov A., Baranov A., Savkin A., Ivanonv M., Calliari L., Passerone R., Karpov E., Suchkov A. (2012). Energy-aware gas sensing using wireless sensor networks. Wireless Sensor Networks.

[3] Razzaque M.A., Akhataruzzaman Adnan M., Abdullah A.H. Energy-Efficient Gas Emission Monitoring Systems Using Wireless Sensor Networks. Proceedings of the Fifth International Conference on Ubiquitous and Future Networks (ICUFN).

[4] Tsow F., Forzani E., Rai A., Rui W., Tsui R., Mastroianni S., Knobbe C., Gandolfi A.J., Tao N.J. (2009). A Wearable and Wireless Sensor System for Real-Time Monitoring of Toxic Environmental Volatile Organic Compounds. IEEE Sens. J..

[5] Popoola O.A.M., Stewart G.B., Landshoff P., Calleja M., Havesb M., Baldov J.J., McLeod M.W., Hodgson T.F., Dicks J., Lewis A. (2013). The Use of Electrochemical Sensors for Monitoring Urban Air Quality in Low-Cost, High-Density Networks. Atmos. Environ..

[6] Wikipedia Foundation; ATEX European Directives Summary. https://en.wikipedia.org/wiki/ATEX_directive.

[7] Manes G., Fusco R., Gelpi L., Manes A., di Palma D., Collodi G. (2012). A Wireless Sensor Network for Precise Volatile Organic Compound Monitoring. Int. J Distrib. Sens. Netw..

[8] Banks A., Gupta R. MQTT Version 3.1.1 Specifications. http://docs.oasis-open.org/mqtt/mqtt/v3.1.1/csprd02/mqtt-v3.1.1-csprd02.html.

[9] Manes G., Fantacci R., Chiti F., Ciabatti M., Collodi G., di Palma D., Manes A. Energy-Efficient MAC Protocols for Wireless Sensor Networks Endowed with Directive Antennas: A Cross-Layer Solution. Proceedings of the IEEE Radio and Wireless Conference.

[10] Alphasense Ltd. Technical Specifications; Doc. Ref. PID-AH/MAR11. http://www.alphasense.com/WEB1213/wp-content/uploads/2014/09/PID-AH.pdf.

[11] Price J.G.W., Fenimore D.C., Simmonds P.G., Zlatkis A. (1968). Design and Operation of a Photoionization Detector for Gas Chromatography. Anal. Chem..

[12] Locke D.C., Meloan C.E. (1965). Study of the Photoionization Detector for Gas Chromatography. Anal. Chem..

[13] Tsujita W., Ishida H., Moriizumi T. Dynamic Gas Sensor Network for Air Pollution Monitoring and its Auto-Calibration. Proceedings of the 2004 IEEE Sensors.

[14] Brunelli D., Minakov I., Passerone R., Rossi M. POVOMON: An Ad-hoc Wireless Sensor Network for Indoor Environmental Monitoring. Proceedings of the IEEE Workshop on Environmental Energy and Structural Monitoring Systems (EESMS).

[15] Brunelli D., Rossi M. (2014). Enhancing lifetime of WSN for natural gas leakages detection. Microelectron. J..

[16] Razzaque M.A., Dobson S. (2014). Energy-Efficient Sensing in Wireless Sensor Networks Using Compressed Sensing. Sensors.

[17] Manes G., Fantacci R., Chiti F., Ciabatti M., Collodi G., di Palma D., Manes A. Enhanced System Design Solutions for Wireless Sensor Networks applied to Distributed Environmental Monitoring. Proceedings of the 32nd IEEE Conference on Local Computer Networks.

[18] Diamond D., Coyle S., Scaramagni S., Hayes J. (2008). Wireless Sensor Networks and Chemo-/Biosensing. Chem. Rev..

[19] Somov A., Baranov A., Spirjakin D., Spirjakin A., Sleptsov V., Passerone R. (2013). Deployment and evaluation of a wireless sensor network for methane leak detection. Sens. Actuators A Phys..

[20] Somov A., Baranov A., Spirjakin D. (2014). A wireless sensor-actuator system for hazardous gases detection and control. Sens. Actuators.

[21] Liu X., Cheng S., Liu H., Hu S., Zhang D., Ning H. (2012). A Survey on Gas Sensing Technology. Sensors.

[22] Jian R.S., Huang Y.S., Lai S.L., Sung L.Y., Lu C.J. (2013). Compact instrumentation for a u-GC for real time analysis of sub-ppb VOC mixtures. Microchem. J..

[23] Sen A., Albarella J.D., Carey J.R., Kim P., McNamara W.B. (2008). Low-cost colorimetric sensor for the quantitative detection of gaseous hydrogen sulfide. Sens. Atuators.

[24] Nakata S., Akakabe S., Nakasuji M., Yoshikawa K. (1996). Gas Sensing Based on a Nonlinear Response: Discrimination between Hydrocarbons and Quantifications of Individual Components in Gas Mixture. Anal. Chem..

